# Association between arginine vasopressin receptor 1A (*AVPR1A*) polymorphism and inequity aversion

**DOI:** 10.1098/rspb.2023.0378

**Published:** 2023-06-14

**Authors:** Hiroki Tanaka, Kuniyuki Nishina, Qiulu Shou, Hidehiko Takahashi, Masamichi Sakagami, Tetsuya Matsuda, Miho Inoue-Murayama, Haruto Takagishi

**Affiliations:** ^1^ Brain Science Institute, Tamagawa University, Tokyo, 194-8610, Japan; ^2^ Graduate School of Brain Sciences, Tamagawa University, Tokyo, 194-8610, Japan; ^3^ Graduate School of Human Sciences, Osaka University, Osaka, 565-0871, Japan; ^4^ Graduate School of Medical and Dental Sciences, Tokyo Medical and Dental University, Tokyo, 113-8519, Japan; ^5^ Wildlife Research Center, Kyoto University, Kyoto, 606-8203, Japan

**Keywords:** inequity aversion, economic game, genetic polymorphism, oxytocin receptor, arginine vasopressin receptor 1A, opioid receptor mu 1

## Abstract

Although numerous studies have focused on brain functions related to inequity aversion, few have examined its genetic basis. Here, we show the association between estimated inequity aversion and polymorphisms in three genes associated with human sociality. Non-student adult participants took part in five economic game experiments on different days. Disadvantageous inequity aversion (DIA) and advantageous inequity aversion (AIA) were calculated from behavioural responses using Bayesian estimation. We investigated the association between genetic polymorphisms in the oxytocin receptor (*OXTR* rs53576), arginine vasopressin receptor 1A (*AVPR1A* RS3) and opioid receptor mu 1 (*OPRM1* rs1799971) and inequity aversion. Regarding *AVPR1A* RS3, participants with the SS genotype had higher AIA than those with the SL or LL genotypes, but no association was found for DIA. Moreover, we observed no aversion associations for *OXTR* rs53576 or *OPRM1* rs1799971. The results suggest that *AVPR1A* plays an important role in aversion when one's own gain is greater than that of others. Our findings may provide a solid theoretical basis for future studies on the relationship between genetic polymorphisms and inequity aversion.

## 1. Introduction

Aversion to unfair distribution is observed among humans not only in developed countries and societies but also in traditional societies [1–4]. These tendencies are expressed in the form of behaviours, such as punishment of norm violators and altruism towards others, and they play a crucial role in achieving and maintaining a cooperative human society [5–7]. Decision-making in response to unfair distribution is modelled by the social preference for inequity aversion [8], which has been examined in numerous studies [9–16]. Inequity aversion consists of two types: disadvantageous inequity aversion (DIA), an aversion to situations where others receive more than you, and advantageous inequity aversion (AIA), where you receive more than others. DIA has appeared among children as young as 3 years of age, while AIA does not develop until around 8 years of age and is strongly influenced by the social environment [17,18].

Over recent years, the biological mechanisms behind inequality aversion, mainly in relation to brain function, have attracted increasing attention. Previous neuroimaging studies have found that inequity aversion is associated with emotion- and value-related brain areas, such as the ventromedial prefrontal cortex, anterior insula, amygdala and striatum [12,14,16,19–23]. Although much research has been conducted on the association between inequity aversion and brain function, little has been conducted on the related genetic factors. One study examined the relationships between inequity aversion and oxytocin receptor (*OXTR*) and gamma-aminobutyric acid-related genes [24], but no other related studies have been conducted. Since inequity aversion is not a human-specific preference [25–29], DIA and AIA have possibly been endowed in humans and other animals through evolution. Therefore, examining the genetic basis underlying DIA and AIA would enhance our understanding of how humans have evolved to form cooperative societies.

Several gene candidates may be associated with preferences for inequality aversion. For example, *OXTR* and genes involved in the receptor for the same neuropeptide, including arginine vasopressin receptor 1A (*AVPR1A*) and opioid receptor mu 1 (*OPRM1*), a receptor for pain-relieving substances, such as beta-endorphins, have important roles in human social behaviour and social emotions [30]. *OXTR*, which encodes a protein associated with the oxytocin receptor, is located at 3p25.3 on chromosome 3. Many single-nucleotide polymorphisms (SNPs) have been identified in *OXTR*, and much attention has been focused on the polymorphism rs53576 in intron 3 and its association with various social traits. Results have shown that people with the GG genotype of rs53576 are more empathetic and more likely to trust others than those with the AA/AG genotypes [31–33].

*AVPR1A*, which encodes the arginine vasopressin V1a receptor, is located at 12q14.2 on chromosome 1. *AVPR1A* has a polymorphism associated with various numbers of tandem repeats in the promoter region, repeating two bases of (GT)_25_, a complex repeat of (CT)_4_-TT-(CT)_8_-(GT)_24_ (RS3) and repetition of the four nucleotide sequences of (GATA)_14_ (RS1). Previous studies have shown that when RS3 is divided into Long (L) and Short (S), a person with the LL genotype is more altruistic in the Dictator Game (DG) than a person with the SS genotype [34]. However, another study showed the opposite result in children with alleles corresponding to L (334 bp allele), who showed more selfish allocation in the DG than those with other allele types [35]. *OPRM1*, which encodes the mu-opioid receptor, is located at 6q25.2 on chromosome 6. Opioids, such as β-endorphin and encephalin, bind to the mu-opioid receptor and show analgesic action. Whereas the A118G (rs1799971) polymorphism in exon 1 of *OPRM1* has long been reported to be associated with individual differences in physical pain sensitivity in palliative medicine, recent studies have demonstrated that it is associated with pain in social situations. Previous studies have shown that people with the G allele at A118G (GG/AG) are more sensitive to social rejection than are people with the AA genotype, and that brain areas involved in emotion, such as the anterior cingulate cortex and the anterior insula, are more strongly activated [36]. However, it remains unclear whether *AVPR1A* or *OPRM1* is associated with inequity aversion.

The present study aimed to examine the association between polymorphisms in three genes (*OXTR* rs53576, *AVPR1A* RS3 and *OPRM1* rs1799971) associated with human sociality in previous studies and inequity aversion. Specifically, DIA and AIA were Bayesian-estimated by a model-based approach using the inequality aversion model [8]. Rather than estimating inequity aversion from behaviour in one particular economic game, such as the Ultimatum Game (UG) [37], this study estimated inequity aversion in a broader context from behavioural indicators in various economic games, including the Prisoner's Dilemma Game (PDG), the DG, the Public Goods Game (PGG), the Trust Game (TG) and the UG.

## 2. Methods

### (a) Participants

This study involved a secondary analysis of data from a large database constructed in a previous project. The research project has collected behavioural and psychological indicators, including behaviour in major economic games, magnetic resonance imaging and genetic polymorphism data. We first analysed 443 (224 female, mean age ± s.d. = 40.8 ± 10.5) subjects who played all five economic games to estimate DIA and AIA. Data from 420 (212 female, mean age ± s.d. = 41.0 ± 10.5) of these subjects with *OXTR*, *AVPR1A* and *OPRM1* genetic polymorphism data were used in the analysis of the association between the estimates and each genotype. Twenty-three individuals were excluded because they did not have both data on the five economic games and genetic data. All experimental protocols were reviewed and approved by the ethics review committee at Tamagawa University. All participants were fully informed of the nature of the study and its possible outcomes, and they provided written informed consent before the study was conducted.

### (b) Estimation of inequity aversion parameters

We estimated the parameters of inequity aversion from the behaviours in the following five economic games: cooperation in the PDG, cooperation in the PGG, allocation in the DG, first-player trust and second-player return rate in the TG, and allocation and rejection in the UG. These tasks were conducted under complete anonymity. The DG was conducted from 27 April to 22 June 2013. The PDG and PGG were conducted from 2 September to 26 October 2013. The TG and UG were conducted from 16 December 2013 to 23 February 2014. The economic games had different measurement dates to minimize carryover effects (electronic supplementary material, figure S1).

### (c) Dictator Game

Participants acted as dictators, deciding how much of the endowment (JPY 1000) they received from the experimenter to provide to an anonymous partner (recipient) in JPY 100 increments. Next, the same game was played six times with different endowment amounts (JPY 300, JPY 400, JPY 600, JPY 700, JPY 1200 and JPY 1300). The proportion of the endowment provided to the recipient in these seven games was used in the analysis.

### (d) Prisoner's Dilemma Game

Participants played a one-shot PDG with an anonymous, randomly assigned partner. Participants decided how much of their endowment (JPY 1000) received from the experimenter to provide to their partner in JPY 100 increments. The paired partner received twice the amount of money provided. For example, if Player A provided JPY 200 and Player B provided JPY 500 to each other, Player A's payoff would be JPY 800 left on hand + (JPY 500 × 2) received from the opponent = JPY 1800. Player B's payoff would be JPY 500 left on hand + (JPY 200 × 2) received from the opponent = JPY 900. The proportion of the endowment provided to the opponent was used in the analysis.

### (e) Public Goods Game

The instructions for the PGG were written assuming a group of 10 players, but participants were told that the actual group size could vary. Participants decided how much of their endowment (JPY 1000) received from the experimenter to provide to the group's public good in JPY 100 increments. The total amount provided was multiplied by two and distributed equally among all group members, independent of the amount provided by each individual. The game was played only once. The proportion of the endowment provided to the public good was used in the analysis.

### (f) Trust Game

The TG is a game played by a truster–trustee pair. The truster decided how much of the endowment (JPY 1000) they received from the experimenter to transfer to the trustee in JPY 100 increments. The trustee took three times the amount transferred by the truster and decided how much to return. Participants decided first as a truster, then as a trustee with another partner, using the strategy method. That is, for all possible patterns of amounts that the truster might offer (JPY 100, JPY 200, JPY 300, JPY 400, JPY 500, JPY 600, JPY 700, JPY 800, JPY 900 and JPY 1000), the participants decided how much of the triple amount to return to the truster in 10% increments. The proportion of the endowment that participants chose to transfer as a truster and the proportion of the amount of money that the participants decided to return as a trustee were used in the analysis.

### (g) Ultimatum Game

Participants first made decisions as proposers, then changed partners and made decisions as responders. As proposers, participants decided on how much of the endowment (JPY 1500) they received from the experimenter to offer to their partners (responders) in JPY 100 increments. As responders, they decided to accept or reject 16 different offers (ranging from JPY 0 to JPY 1500) made by their partners (proposers) using the strategy method. Both proposer and responder decisions were used in the analysis. The proportion of the endowment offered to the partner (as proposer) and the overall decision of whether to accept or reject each of the 16 possible offers (as responder) were used.

### (h) Modelling

Participants’ behaviours were modelled using the inequity aversion model [8], which can explain economic decision-making according to unwillingness for unequal resource distribution. In this model, the player's utility for a given distribution between self and other, U(x,y), is represented by the following utility function:

$$U(x,y)=x-\alpha \left(\max\left(\frac{x+y}{2}-x,0\right)\right)-\beta \left(\max\left(x-\frac{x+y}{2},0\right)\right).$$

The *x* and *y* represent the payoffs that the self and the other receive from the resource distribution, respectively. The second term on the right-hand side represents the magnitude of inequality when the other gains more. The third term on the right-hand side represents the magnitude of the inequality when the self-gains more. These are weighted by two parameters, α (or DIA) and β (or AIA), respectively, which represent the unwillingness to accept an unequal distribution. For example, the greater α, the more the player dislikes a situation in which she/he is at a relative disadvantage to the opponent, no matter how large her/his absolute payoff would be. By contrast, the greater β, the more the player is concerned about whether she/he would earn too much compared to the opponent. Therefore, the inequity aversion model discounts the utility of a given distribution depending on its deviation from equality and depending on one's aversion to that deviation.

The conversion of each utility into the probability of proposing (or accepting) the distribution is modelled by the following softmax function:

$$P(x^{\prime },y^{\prime })=\frac{\exp(\lambda U(x^{\prime },y^{\prime }))}{\sum \exp(\lambda U(x,y))}.$$

Here, (x,y) means the set of all possible distributions in each game, whereas $\left(x^{\prime },y^{\prime }\right)$ means proposing (accepting) the distribution among (x,y). λ represents the softmax inverse temperature parameter. The lower this value, the closer the decision is to random choice. Using Bayesian inference with Markov chain Monte Carlo methods, we estimated three parameters (α, β and λ) for each participant by fitting these models to the behavioural data in all games.

### (i) Genotyping

Participants’ buccal mucosa cells were collected between 25 October 2014 and 25 January 2015. DNA was extracted using the DNeasy Blood & Tissue Kit (QIAGEN, Tokyo, Japan) according to the manufacturer's protocol. Genotyping of *OXTR* rs53576 was conducted using the loop-mediated isothermal amplification (LAMP) Genotyping Series Human *OXTR* (rs53576; Nippon Gene, Toyama, Japan) by mixing fluorescently labelled LAMP primers and *Bst* DNA polymerase. The fluorescence level of the reactant was measured by the LAMP-FLP method using a Genie II (Nippon Gene). The same protocol was used in our previous study [33].

Genotyping of *OPRM1* A118G was conducted using the same protocol as for *OXTR* rs53576; only the *OPRM1* A118G fluorescently labelled LAMP primers were different. Regarding the genotyping of *AVPR1A*, to amplify the microsatellite polymorphism in the RS3 located on the promoter region, the DNA product was amplified using polymerase chain reaction (PCR). We used the primers 5′-FAM-TCCTGTAGAGATGTAAGTGC-3′ (forward) and 5′-TCTGGAAGAGACTTAGATGG-3′ (reverse) [38–40].

PCR amplification was performed under the following conditions: 94°C for 1 min, followed by (94°C for 30 s, 60°C for 30 s, 74°C for 1 min) × 35 cycles, and a final extension at 74°C for 10 min. The PCR products were analysed using an ABI 3130xl DNA Sequencer and GeneMapper Software (Applied Biosystems, Foster City, CA, USA). Following a previous study [41], the median of the allele distribution was used as the threshold to ensure that the number of participants in the S and L categories was in the same proportion. We defined the allele shorter than the median (less than 330) as 'short' and the allele longer than the median (greater than or equal to 330) as 'long'. All participants were classified into three groups (SS, SL and LL) according to the length of their allele. Notably, some studies have targeted the 334 bp allele [35,40], a risk allele in *AVPR1A*. Therefore, this study also examined the association between the 334 bp allele and the inequity aversion parameters.

### (j) Statistical analysis

All analyses reported in this paper were performed using SAS 9.4 (https://www.sas.com/) and RStudio 1.2.5033 (https://posit.co/blog/rstudio-new-open-source-ide-for-r/). To estimate each participant's inequity aversion preferences (DIA and AIA), we used the RStan package (version 2.19.3; https://mc-stan.org/users/interfaces/rstan) to perform Bayesian inference. To examine the relationship between the estimates and the polymorphisms, we performed an analysis of covariance (ANCOVA) with DIA and AIA as dependent variables, three gene polymorphisms (*OXTR*, *AVPR1A* and *OPRM1*) as independent variables and age as a covariate. The reason for adding age as a covariate is that age was correlated with inequity aversion in the prior analysis (DIA: *r* [418] = 0.13, *p* = 0.007, AIA: *r* [418] = 0.32, *p* < 0.001). Income, one of the indicators of socioeconomic status, was not included as a covariate because it was not associated with the inequity aversion parameters (DIA: *F*_6,408_ = 0.85, *p* = 0.534, *pη*^2^ = 0.012; AIA: *F*_6,408_ = 1.04, *p* = 0.400, *pη*^2^ = 0.015). When a main effect of genetic polymorphism was observed, multiple comparisons between genotypes were performed using a Bonferroni-corrected *p*-value (corrected *p*-value = 0.017) based on the number of genotypes (three genotypes). Because sex differences are well documented for oxytocin and vasopressin [33,42–45], interaction effects between sex and genetic polymorphisms were examined in additional analyses. Since the interaction effects between sex and genetic polymorphisms were not explicitly predicted, the analysis results are presented in the electronic supplementary material.

## 3. Results

### (a) Genotype distribution

Electronic supplementary material, figure S2 shows the proportions of genotypes for each gene. The proportion of *OXTR* rs53576 genotypes was 40.7% (*n* = 171) for AA, 46.7% (*n* = 196) for AG and 12.6% (*n* = 53) for GG. The proportion of *AVPR1A* RS3 genotypes was 27.9% (*n* = 117) for SS, 48.3% (*n* = 203) for SL and 23.8% (*n* = 100) for LL. The proportion of *OPRM1* rs1799971 genotypes was 27.1% (*n* = 114) for AA, 47.9% (*n* = 201) for AG and 25.0% (*n* = 105) for GG. No association of genotype proportions was found for any of the genes (*OXTR* and *AVPR1A*: *χ*^2^ = 4.49, *p* = 0.344, electronic supplementary material, table S1; *OXTR* and *OPRM1*: *χ*^2^ = 2.04, *p* = 0.728, electronic supplementary material, table S2; *OPRM1* and *AVPR1A*: *χ*^2^ = 5.04, *p* = 0.284, electronic supplementary material, table S3). These distributions were not significantly different from Hardy–Weinberg equilibrium (*OXTR*: $\chi_{1}^{2}=0.075$, *p* = 0.785; *AVPR1A*: $\chi_{1}^{2}=0.423$, *p* = 0.515; *OPRM1*: $\chi_{1}^{2}=0.756$, *p* = 0.385). In addition, the proportion of carriers of the 334 bp allele in *AVPR1A* RS3 was 36.7% (*n* = 154), and that of non-carriers was 63.3% (*n* = 266).

### (b) Inequity aversion parameters and game behaviours

Electronic supplementary material, figure S3 shows the relationship between estimated DIA and AIA and behaviour in each game. DIA was positively correlated with rejection behaviour in the UG when one's distribution was smaller than that of others. By contrast, AIA was positively correlated with behaviour in games measuring so-called prosocial behaviour, such as the DG, PGG, PDG, TG and proposer's behaviour in UG. Two estimated parameters (DIA and AIA) were positively correlated (*r* [418] =0.16, *p* = 0.0007).

### (c) Disadvantageous inequity aversion

**Figure 1.**
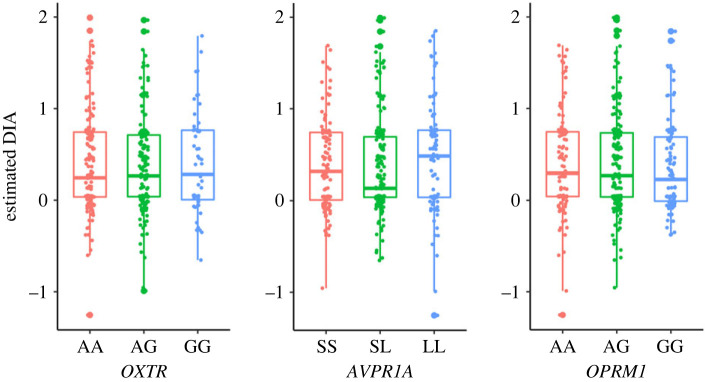
Relationships between disadvantageous inequity aversion (DIA, α parameter) and genotypes.

The mean levels of DIA for each genetic polymorphism are shown in figure 1. ANCOVA results showed no effect of any of the *OXTR* (*F*_2,412_ = 0.29, *p* = 0.747, p*η*^2^ = 0.001), *AVPR1A* (*F*_2,412_ = 1.46, *p* = 0.234, p*η*^2^ = 0.007) and *OPRM1* (*F*_2,412_ = 0.21, *p* = 0.807, p*η*^2^ = 0.001) polymorphisms on DIA. No interaction effect between sex and genetic polymorphisms was observed when sex was added as a factor in the analysis (electronic supplementary material, figure S4*a*). Even when the effect of *AVPR1A* was examined using the 334 bp allele rather than the allele's length, the 334 bp allele had no effect (*F*_1,413_ = 0.01, *p* = 0.935, *pη*^2^ < 0.001) (electronic supplementary material, figure S5*a*).

### (d) Advantageous inequity aversion

**Figure 2.**
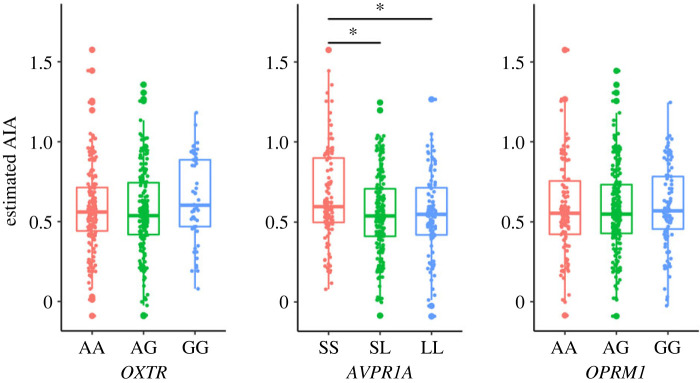
Relationships between advantageous inequity aversion (AIA, β parameter) and genotypes. *p_corrected_ < 0.05.

The mean AIA levels for each genetic polymorphism are shown in figure 2. ANCOVA results showed an effect of *AVPR1A* (*F*_2,412_ = 4.80, *p* = 0.009, p*η*^2^ = 0.023) on AIA, but no effect of *OXTR* (*F*_2,412_ = 1.83, *p* = 0.162, p*η*^2^ = 0.009) and *OPRM1* (*F*_2,412_ = 0.06, *p* = 0.939, p*η*^2^ < 0.001). For *AVPR1A*, multiple comparisons showed that participants with the SS genotype had higher AIA than those with the SL genotype (*t*[412] = 2.9, *p* = 0.004, *d* = 0.34) or those with the LL genotype (*t*[412] = 2.5, *p* = 0.012, *d* = 0.35). No significant differences were observed between participants with the SL genotype and those with the SS genotype (*t*[412] = 0.05, *p* = 0.964, *d* = 0.006). The results of the analysis, in which sex was added as a factor, revealed no interaction effect between any of the gene polymorphisms and sex (electronic supplementary material, figure S4*b*). The effect of *AVPR1A* was observed when using the 334 bp allele rather than the allele's length (*F*_1,413_ = 8.38, *p* = 0.004, p*η*^2^ = 0.020); AIA was lower in participants with the 334 bp allele than in those without (electronic supplementary material, figure S5*b*).

## 4. Discussion

This study showed that AIA tended to be higher in people with the SS genotype than in those with the SL and LL genotypes of *AVPR1A*. AIA can be considered a prosocial preference because it is the dislike one feels when one receives more rewards than others. In fact, AIA was positively correlated with prosocial behaviour in the economic games in the current study. The association of *AVPR1A* with prosocial behaviour has been reported in previous studies [34,35]. The study by Knafo *et al*. [34] examined the association between allocation in the DG and *AVPR1A* RS3 polymorphisms and found that people with SS genotypes were less altruistic than those with other genotypes. By contrast, Avinun *et al*. [35] found that the 334 bp allele carriers (corresponding to the L allele in the Knafo *et al*. study) were less altruistic in the DG than were non-carriers. These results suggest that when *AVPRA1* RS3 is divided into S and L, there is an effect of the L allele on AIA, with a strong effect of the 334 bp allele in those with L. One reason for the inconsistent association between *AVPR1A* RS3 and altruism may be the instability of the DG. The DG is highly dependent on the experimental situation owing to the simplicity of the task [46,47]. To avoid such instability, this study used behavioural data from multiple economic games to estimate people's prosociality. The present study showed an association between more pervasive prosocial behaviour and *AVPR1A* in different situations rather than in a single economic game situation. In the future, it will be important to conduct such a multi-task study to show more reliable results.

*AVPR1A* encodes the vasopressin V1a receptor and is involved in vasoconstriction. In the central nervous system, it acts by binding to vasopressin in subcortical regions, such as the limbic system, and promotes amygdala activity [48]. Meyer-Lindenberg *et al*. [48] have shown that people with the L allele exhibit amygdala hyperactivity in response to fearful faces. These results suggest that V1a receptors are more abundantly distributed in the amygdala of L-allele carriers and that the amygdala is hyperactive in response. In economic decision-making, people tend to be sensitive to financial loss, which is known as loss aversion. Previous studies have shown that the amygdala has a pivotal role in loss aversion [49,50]. De Martino *et al*. [50] found that participants with bilateral focal amygdala lesions had lower loss aversion. In addition, Canessa *et al*. [49] found that activity in the amygdala and posterior insula was associated with the magnitude of financial loss and that the degree of loss aversion was associated with grey matter volume in the amygdala. Therefore, the results of the current study suggest that people with the LL and SL genotypes of *AVPR1A* have a stronger V1a receptor action in the amygdala than those with the SS genotype, resulting in a higher loss aversion tendency. Since high loss aversion tendencies lead to keeping one's own money, levels of prosocial behaviour may have been lower in those with the L allele. Another possibility is the anxiogenic effect of arginine vasopressin [51,52]. For example, people with the SS genotype in *AVPR1A* RS3 may have exhibited AIA because of the strong effects of arginine vasopressin, which arouses anxiety in social situations, such as when there are reputation concerns.

Unlike *AVPR1A*, *OXTR* and *OPRM1* showed no association with AIA. Given the previous *OXTR* studies, this lack of association with AIA is surprising. However, the oxytocin system has the role of regulating attitudes and beliefs, which are antecedents of prosocial behaviour [33,53]. Although previous human and animal studies have shown that the oxytocin system undoubtedly has an anxiety-buffering effect, whether it directly affects the AIA estimated in this study is another matter. In recent years, the effect of oxytocin administration on actual behaviour has been questioned [54], suggesting that it is indirectly, rather than directly, related to prosocial behaviour through anxiety buffering and other mechanisms. Additionally, betrayal aversion acts as a factor inhibiting prosocial behaviour in more prosocial individuals, as measured by social value orientation, but it is not associated with prosocial behaviour in pro-self individuals [55]. This suggests that the process by which prosocial behaviour arises strongly differs between individuals. These individual differences in the association between anxiety buffering and prosocial behaviour may explain why we found no association between *OXTR* and AIA. As the social salience hypothesis also proposes that oxytocin modulates sensitivity to social stimuli [56], oxytocin is thought to play a pivotal role in the process of generating prosocial behaviour. Another reason for the lack of association between *OXTR* and AIA may be the small number of people with the GG genotype. Previous studies have shown that East Asians have an extremely low number of GG genotypes compared with North Americans [57,58]. In this study, as in previous studies, the number of people with the GG genotype was small (12% of all participants). If there are extreme cultural differences in the distribution of genotypes, we believe that it is necessary to collect data from people with more than one cultural background.

Although the polymorphism in *AVPR1A* was only associated with AIA and not DIA, this result is not unreasonable. In the inequity aversion model, DIA and AIA are free parameters introduced to distinguish which aspects of inequity (disadvantageous or advantageous to oneself) are focused on. This means that neither a negative nor a positive direction of association between DIA and AIA is assumed. In real life, there may be people who are averse to situations where the other party is benefiting more than them (i.e. high DIA), while feeling no aversion to situations where they gain more (i.e. low AIA) or *vice versa* (i.e. low DIA and high AIA). However, there may be people who are averse to unequal distribution itself, regardless of whether they or the opponent gains more (i.e. high DIA and high AIA) as well as people who do not have any aversion to inequality (i.e. low DIA and low AIA). Therefore, the decision not to make an assumption about the correlation between DIA and AIA may be valid. Moreover, several previous studies have reported that one of these parameters is significantly associated with a particular variable, while the other is not [12,14]. Although DIA and AIA were significantly correlated in the current study, the effect size is not large (*r* = 0.16). Therefore, there is no theoretical or empirical support for the assertion that a variable associated with AIA (or DIA) should necessarily be associated with DIA (or AIA) also.

Nevertheless, the current result that no association was found between DIA and any of the polymorphisms in *OXTR*, *AVPR1A* or *OPRM1* is surprising in some aspects. Previous studies have shown that AIA development is not expressed until around 8 years of age and is influenced by the social environment, whereas DIA is observed at earlier developmental stages [17,18]. Furthermore, DIA has been observed not only in humans, but also in several primates, dogs and some birds, such as crows [25–29]. If traits seen earlier in development or in other species are more likely to be innate in humans, DIA is more likely to be caused by certain genetic factors than is AIA. This contradiction may be due to the limited targets of the genetic polymorphisms examined in the current research. For example, other SNPs in *OXTR* besides rs53576 are also associated with prosociality [59–61]. Additionally, for *AVPR1A*, associations between polymorphisms other than RS3 and prosociality have been reported [62,63]. Certainly, it is possible that genes and genetic polymorphisms not yet examined are associated with inequity aversion, and that the preference is not necessarily defined by a single genetic polymorphism. Adopting an approach that analyses multiple polymorphisms together would enable a more detailed examination of the contribution of genes to inequity aversion.

This study had some limitations that have to be taken into consideration in future studies. First, whether the L allele suppressed AIA or the S allele promoted AIA remains unclear. To answer this question, how neuroscientific substrates mediate the relationship between the *AVPR1A* genotype and AIA needs to be addressed. We consider the mediating role of the amygdala, where the expression of *AVPR1A* has been demonstrated in mice [64]. People with the L allele of this gene show higher activity when performing an anger or fear face-matching task [48]. In reference to levels of neuropeptide, arginine vasopressin enhances neural activity in the amygdala [65] and is associated with anxiety-related behaviour [66]. These studies suggest that the amygdala mediates the association between the L allele of *AVPR1A* and lower AIA. Further studies are necessary to clarify this gene–brain preference relationship. Second, we examined the genetic effect on inequality aversion only by analysing the association between individual preferences and three polymorphisms of specific genes (*AVPR1A, OXTR* and *OPRM1*) that have been associated with prosociality. This approach may lead to limited conclusions. To more thoroughly determine the genetic basis underlying inequality aversion, we advise future researchers to use different approaches, such as twin studies [67], to analyse the degree of the genetic effect on inequality aversion. Third, although we observed that *AVPR1A* was associated with AIA, whether *AVPR1A* is associated specifically with AIA or also with other aspects of prosociality remains unclear. For example, several researchers have focused on reciprocity (meeting a reciprocal expectation of another's cooperation), which is an important factor in the decision process of human prosocial behaviour as well as inequity aversion [68–71]. By using a modified version of the TGs, previous studies measured participants' prosocial motivations separately as inequity aversion and reciprocity and found neural correlates for each of them. This means that the separation of inequity aversion and reciprocity motives requires limiting the context of the game. Since the main interest of the current study was inequity aversion as a common motivation for social decision-making observed through multiple contexts of economic games (i.e. DG, PDG, PGG, TG and UG), the restriction of the social context for the separation of motives was not possible. It is important to note that at least the AIA estimated in the current study was not exclusive to the reciprocity motive but inclusive owing to using such multiple contexts of the games. Therefore, whether *AVPR1A* is only associated with the aversion to the difference in self- and other-payoff, or with an aversion to disappointing others’ expectations simultaneously, requires further investigation. Fourth, the strategy method was used. The strategy method is effective in experiments conducted without using deception in economic games. In order to avoid using deception in the present study, the strategy method was used. However, methods that do not use the strategy method are more likely to elicit emotional responses because they allow us to examine the partner's actual reactions to the behaviour. If so, the association between *AVPR1A* and inequity aversion may be stronger in experimental designs that do not use the strategy method. Such studies are encouraged in the future. Finally, the reproducibility of this study is mentioned. In the case of exploratory studies such as the present study, at least one of the following characteristics should be met: (i) report direct replication experiments with sufficient power in the same paper; (ii) report the results of analyses with appropriate statistical corrections; and (iii) report all analyses performed on the same dataset [72]. Although this study does not report direct replication experiments, the results of the analysis with appropriate corrections (based on the number of genotypes) and all analyses are reported in the study. Therefore, this study meets the criteria for a genetic polymorphism study. Direct replication experiments need to be performed in the future.

The correction of inequality is an important factor not only for good interpersonal relations but also for the sustainability of a co-prosperous society. Therefore, understanding how inequality aversion preferences arise among us and what the genetic basis is for these individual differences is worthwhile. The current results show an association between the *AVPR1A* polymorphism and AIA in humans. This may shed light on the genetic origins of fairness and provide an informed perspective on the more pragmatic question of how we can achieve a society without any inequality.

## Data Availability

Data are available from the Dryad Digital Repository: https://doi.org/10.5061/dryad.1vhhmgqxg [73]. Supplementary material is available online [74].
